# Update: Recommendations for Middle East Respiratory Syndrome Coronavirus (MERS-CoV)

**Published:** 2013-07-12

On June 11, 2013, CDC issued interim infection prevention and control recommendations for hospitalized patients with known or suspected Middle East respiratory syndrome coronavirus (MERS-CoV) infection in U.S. hospitals (1). To date, no MERS-CoV cases have been reported in the United States; however, cases have been reported in eight other countries (2). Recent published reports (3,4) have described limited health-care transmission of MERS-CoV, including cases among health-care personnel in international settings. These published reports highlight the need for rapid detection of infectious patients and adherence to correct infection prevention measures to prevent transmission of the virus among patients, health-care personnel, and visitors.

In coming months, the U.S. health-care system might be called upon to provide care to patients infected with MERS-CoV. Front-line providers and health-care organizations should be prepared to care for MERS-CoV patients as part of routine operations. To aid providers and facilities, CDC has developed checklists that identify key actions that can be taken now to enhance preparedness for treating persons with MERS-CoV infection and compiled a list of preparedness resources (available at http://www.cdc.gov/coronavirus/mers/preparedness).

Additional information, including guidance on case definitions, infection control, case investigation, and specimen collection and testing, is available at the CDC MERS website (2). The MERS website contains the most current information and guidance, which is subject to change. State and local health departments with questions should contact the CDC Emergency Operations Center at telephone, 770-488-7100.
